# Crystal structure of glycogen debranching enzyme and insights into its catalysis and disease-causing mutations

**DOI:** 10.1038/ncomms11229

**Published:** 2016-04-18

**Authors:** Liting Zhai, Lingling Feng, Lin Xia, Huiyong Yin, Song Xiang

**Affiliations:** 1 Key Laboratory of Nutrition and Metabolism, Institute for Nutritional Sciences, Shanghai Institutes for Biological Sciences, Chinese Academy of Sciences, Shanghai 200031, China

## Abstract

Glycogen is a branched glucose polymer and serves as an important energy store. Its debranching is a critical step in its mobilization. In animals and fungi, the 170 kDa glycogen debranching enzyme (GDE) catalyses this reaction. GDE deficiencies in humans are associated with severe diseases collectively termed glycogen storage disease type III (GSDIII). We report crystal structures of GDE and its complex with oligosaccharides, and structure-guided mutagenesis and biochemical studies to assess the structural observations. These studies reveal that distinct domains in GDE catalyse sequential reactions in glycogen debranching, the mechanism of their catalysis and highly specific substrate recognition. The unique tertiary structure of GDE provides additional contacts to glycogen besides its active sites, and our biochemical experiments indicate that they mediate its recruitment to glycogen and regulate its activity. Combining the understanding of the GDE catalysis and functional characterizations of its disease-causing mutations provides molecular insights into GSDIII.

## Abstract (web summary)

Debranching of glycogen is an important step in its use as an energy source. Here, the authors describe the crystal structures of glycogen debranching enzyme alone and in complex with oligosaccharides and provide molecular insights into the function, and into associated diseases.

## Graphical abstract

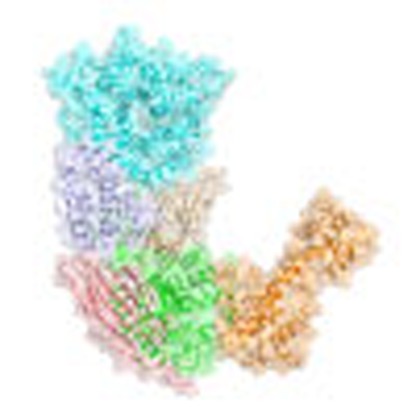

Glycogen is present in organisms from bacteria and archaea to humans, and serves as an important energy store. It is a highly branched glucose polymer, with a branch point occurring at every 12 glucose residues on average^1^. Plants synthesize starch, a mixture of amylopectin and α-amylose. The former differs from glycogen by having fewer branches, and the latter is a liner glucose polymer. In response to energy needs, glucose residues are released from glycogen/starch by glycogen phosphorylase. This process comes to a halt four residues before the branch point, making glycogen/starch debranching an essential step in its mobilization. In humans, this reaction is catalysed by glycogen debranching enzyme (GDE)^1^,^2^. The functional importance of GDE is underscored by the fact that its deficiencies are associated with GSDIII. Patients accumulate abnormally structured glycogen in liver and skeletal muscle, and the clinical symptoms include hypoglycemia, growth retardation, hepatomegaly, hypertrophic cardiomyopathy and progressive skeletal myopathy^3^,^4^.

The glucose residues in glycogen/starch are linked via α-1,4 glycosidic bonds, and α-1,6 glycosidic bonds at branch points. GDE possesses the α-1,4 glucanotransferase (GT) and α-1,6 glucosidase (GC) activities, and removes glycogen phosphorylase-digested branches in two steps^5^,^6^. Its GT activity transfers a maltotriosyl group from the branch to a neighbouring non-reducing end, and forms an α-1,4 glycosidic bond between them. The last residue on the branch is subsequently removed by its GC activity (Supplementary Fig. 1a,b). GDE is highly conserved in animals and fungi^7^. It is distinct from enzymes participating in glycogen/starch debranching in bacteria and plants. These enzymes, including isoamylases and pullulanases, possess only the GC activity, are 50% smaller, and share very limited sequence similarity with GDE. Although the *Sulfolobus solfataricus* enzyme Trex has been reported to possess both GT and GC activities^8^,^9^, it is closely related to these enzymes^10^ and distinct from GDE.

GDE from a number of species have been purified and characterized^11^,^12^,^13^,^14^,^15^,^16^. Its GT and GC activities can be selectively inactivated by limited proteolysis^17^ or inhibitors^18^,^19^, suggesting that they are located at distinct regions. A modest sequence similarity between its N-terminal region and amylases/GTs in the glycoside hydrolase (GH) family 13 has led to the proposal that this region encodes the GT activity^20^. This similarity is largely concentrated at four short conserved sequence regions (CSRI–IV), which contain the catalytic residues in GH13 family members^21^. The functional importance of the equivalent residues in GDE was demonstrated by intermediate trapping experiments^22^ and mutagenesis studies^23^ and the latter also revealed that mutations in the GDE C-terminal region abolished its GC activity.

Although GDE plays a critical role in glycogen metabolism and has been discovered more than 40 years ago, the molecular basis of its function is still poorly understood. This is largely due to a lack of understanding of its three-dimensional structure, despite the crystallization^24^ and cryo-EM studies^25^ of GDE. We report here, the crystal structures of GDE up to 3.1 Å resolution, alone and in complex with oligosaccharides, and structure-guided mutagenesis and biochemical studies to assess the structural observations. Our studies reveal how the GT and GC catalysis proceed and are coordinated in the unique GDE tertiary structure, which provides additional contacts to glycogen that mediate its recruitment to glycogen and regulate its function. In combination with functional characterizations of the GDE disease-causing mutations, these studies provide molecular insights into GSDIII.

## Results

### Structure determination

We expressed *Candida glabrata* GDE (CgGDE, 38% sequence identity to human GDE, Supplementary Fig. 2) in *Escherichia coli* and crystallized it. A multiple wavelength anomalous diffraction (MAD) experiment using a selenomethionine (SeMet)-substituted crystal gave an excellent electron-density map (Supplementary Fig. 3a), which allowed us to build the complete structure (Supplementary Fig. 3b). To study its interaction with glycogen, we also determined a complex structure of CgGDE with maltopentaose by the molecular replacement method. For the complex structure a GT-defective mutant (E564Q, see below) was used to protect maltopentaose from being converted to other oligosaccharides^16^. These structures were refined to resolutions of 3.1 and 3.3 Å, respectively, and agree well with crystallographic data and expected geometric values (Table 1).

Two very similar protomers were found in the ligand-free crystal. Protomer A contains residues 3–1,528, and protomer B contains residues 4–284, 287–330, 336–341, 345–432 and 442–1,528. The root mean square deviation (r.m.s.d.) for their related Cα atoms is 1.1 Å. Residues 629–651 have the largest conformational difference. In protomer A this region adopts a loop structure. In protomer B this region forms a β-hairpin that contains an inter-strand disulfide bond between Cys634 and Cys645, and sticks out of the structure. The orientations of residues 237–458 in these protomers are also different, and are related by a 6° rotation (Supplementary Fig. 3c,d). In the maltopentaose complex crystal there are also two very similar protomers, both containing residues 3–1,528, and the r.m.s.d. for their related Cα atoms is 0.4 Å (Supplementary Fig. 3e). Their structures are very similar to that of protomer A in the ligand-free crystal, the r.m.s.d. for the related Cα atoms are around 0.6 Å (Supplementary Fig. 3f).

### Overall structure of GDE

**Figure 1:**
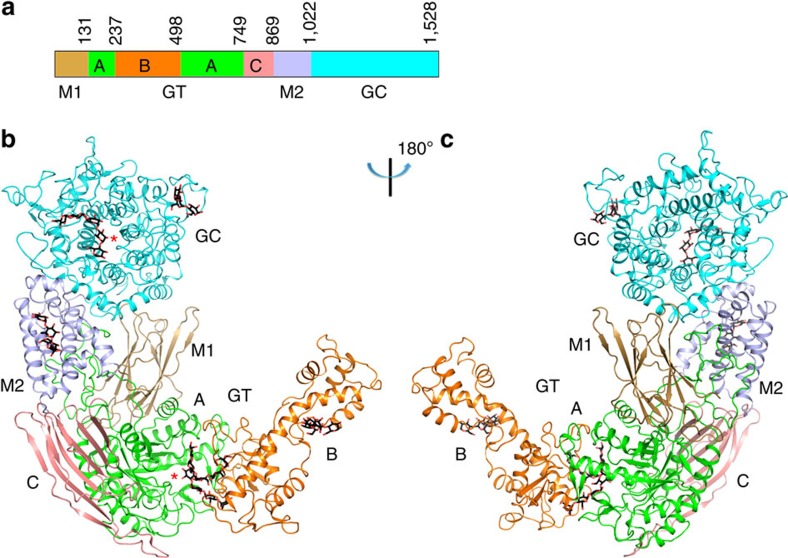
Structure of CgGDE. (**a**) Domain arrangement of GDE. Domains M1, M2 and GC, and the GT subdomains A, B and C are colour-coded. This colouring scheme is used throughout the manuscript unless otherwise indicated. Domain boundaries for CgGDE are indicated. (**b**,**c**) Crystal structure of CgGDE. The views are related by an 180° rotation around the vertical axis. The red stars in **b** indicate the active sites. Bound oligosaccharides are shown in stick representation with their carbon atoms in black. Structural figures were prepared with pymol (http://www.pymol.org).

The structures revealed that CgGDE is composed of four domains, and adopts an elongated structure. Its GT and GC domains (see below) are located on opposite ends of the molecule, with two middle domains (M1 and M2) located in parallel between them (Fig. 1a–c and Supplementary Fig. 3b). Domains GT and GC have little contacts with each other. The middle domains form extensive interactions with them, playing important roles in stabilizing their structure and maintaining the GDE tertiary structure. The interface between them and domain GT buries 7,700 Å^2^ of surface area (6,800 Å^2^ for protomer B in the ligand-free crystal, due to the conformational change of residues 629–651), and that between them and domain GC buries 3,800 Å^2^.

Domains M1 and M2 have little contact with each other and a large cavity exists at their interface. A long loop in domain GT (residues 608–659) protrudes into this cavity and interacts with both domains. A 34-residue stretch in this loop does not exist in human GDE (residues 619–652, Supplementary Figs 2 and 3d). In protomers A and B in the ligand-free crystal the buried surface areas between this stretch and domains M1 and M2 are 2,600 Å^2^ and 1,700 Å^2^, respectively. However the absence of this stretch in human GDE is unlikely to create any significant differences in its tertiary structure, since extensive interactions are mediated by other parts of domain GT, and the large conformational change of residues 629–651 did not cause any significant differences in the CgGDE tertiary structure.

Our dynamic light-scattering experiments indicated CgGDE is monomeric in solution, like GDE in other species^11^,^13^,^14^,^15^. Consistent with this, the interactions between the CgGDE molecules in both crystals are not extensive.

### Molecular insights into the GT and GC catalysis

**Figure 2:**
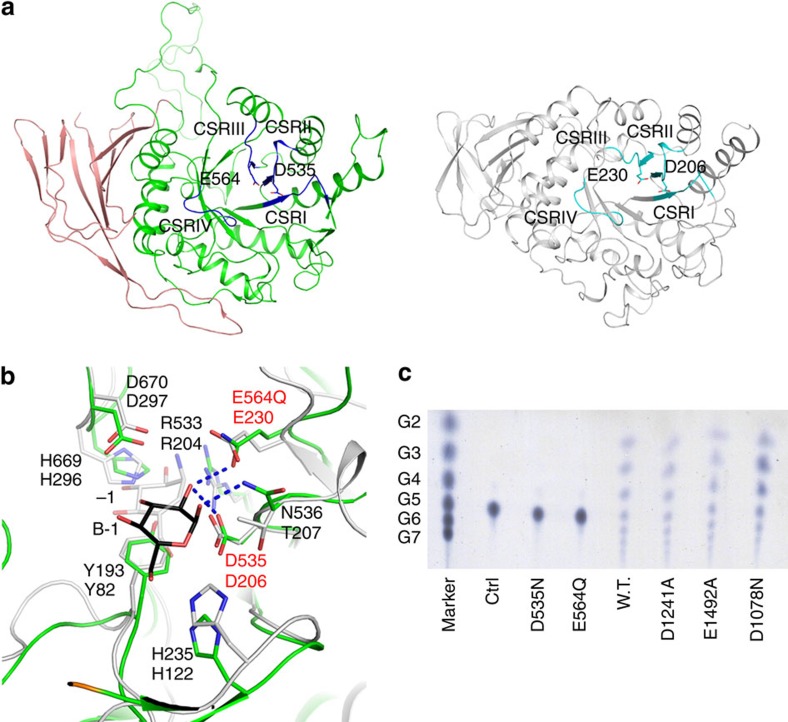
Structure and function of the GT domain. (**a**) Structure of the GT domain. Structure of Taka-amylase A (right, PDB 7TAA) is shown for reference. CSRI–IV in both structures are coloured in blue and cyan, respectively. Catalytic residues are highlighted. Subdomain B and the equivalent region in Taka-amylase A (domain B) are omitted for clarity. (**b**) Structure of the GT domain active-site pocket. The −1 subsite in Taka-amylase A (grey for the carbon atoms) is superimposed for reference. Amino acid residue labels on the second lines are for Taka-amylase A. Catalytic residues are labelled in red. The B-1 residue of the bound oligosaccharide B in the maltopentaose complex structure and the −1 saccharide unit of acarbose in the Taka-amylase A structure are shown. (**c**) GT activity of CgGDE and its mutants. The reactions catalysed by CgGDE and its mutants with maltopentaose as the substrate were analysed with thin-layer chromatography. In the control experiment (lane Ctrl) no CgGDE were added to the reaction. Oligosaccharides with 2–7 residues (G2–G7) were used as standards.

Consistent with the modest sequence similarity between them, the structure of the CgGDE N-terminal region (residues 132–869) is homologous to that of GH13 family members, and CSRI–IV in them occupy similar locations (Fig. 2a). Like GH13 family members, this region can be further divided into three subdomains: a TIM barrel subdomain A followed by an all-β subdomain C, and a subdomain B inserted between β3 and α3 of subdomain A. Subdomains A and C are equivalent to domains A and C in GH13 family members. Subdomain B appears to adopt a novel fold, and the equivalent region in GH13 family members (domain B) is variable. Reactions catalysed by GH13 family members involve an initial cleavage of the glycosidic bond between the +1 and −1 residues of their polysaccharide substrates. They have a highly conserved catalytic core, the −1 subsite, which accommodates the −1 residue and provides the catalytic nucleophile and proton donor (for instance, Asp206 and Glu230 in Taka-amylase A)^21^. Despite the low overall sequence identities between CgGDE and GH13 family members (no more than 15%), the equivalent region in CgGDE adopts an almost identical structure, with Asp535 and Glu564 occupying equivalent locations as the catalytic nucleophile and proton donor, respectively (Fig. 2b).

This homology suggests that the N-terminal region of CgGDE catalyses a GT reaction similar to that of GTs in the family GH13, and we have named it the GT domain. To verify this, we generated the D535N and E564Q mutants and tested them for GT activity. As expected, both mutants completely lost this activity (Fig. 2c). In *Saccharomyces cerevisiae* GDE (ScGDE), the equivalent mutations (D535N and E564Q) were also found to abolish its GT activity^23^. The catalytic role of the Asp535 equivalent has also been demonstrated in rabbit muscle GDE (Asp549)^22^.

**Figure 3:**
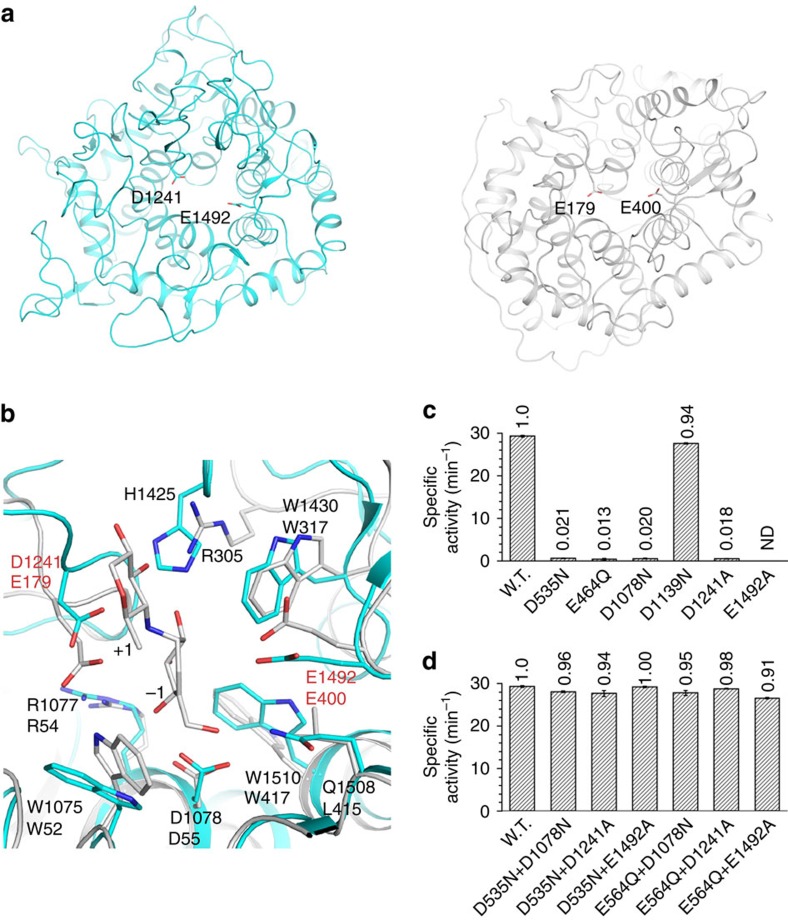
Structure and function of the GC domain. (**a**) Structure of the GC domain. Structure of the *Aspergillus awamori* glucoamylase catalytic domain (right, PDB 1GAH) is shown for reference. Catalytic residues are highlighted. (**b**) Structure of the GC domain active-site pocket. Structure of the *Aspergillus awamori* glucoamylase is superimposed for reference (grey for the carbon atoms). Amino acid residue labels on the second lines are for glucoamylase. Catalytic residues are labelled in red. The +1 and −1 saccharide units of acarbose in the glucoamylase structure are shown. They mimic the +1 and −1 residues in its substrate, the glycosidic bond between which gets hydrolysed. (**c**) Specific debranching activities of CgGDE and its mutants. The specific activity is defined as the debranching reaction rate at the substrate concentration of 13 mg ml^−1^ (the reaction rate of the wild-type CgGDE plateaus at this substrate concentration, see Supplementary Fig. 4a), divided by the concentration of CgGDE. The error bars indicate standard deviations of triplicate experiments. The numbers above each bar indicate ratios to the wild-type value. ND indicates not detected. (**d**) Specific debranching activities of combinations of a GT-defective mutant and a GC-defective mutant. In reactions catalysed by a combination of mutants, they were added in a 1:1 ratio, and the concentration of one is used in calculating the specific activity. The activity of the wild-type CgGDE is shown for reference.

The C-terminal region of CgGDE (residues 1,023–1,528) adopts a (α/α)_6_-barrel structure, homologous to structures of the catalytic domain in glucoamylase (Fig. 3a) and other GH15 family members. These enzymes hydrolyse α-glycosidic bonds at the non-reducing end of polysaccharides, with two conserved acidic residues serving as the general acid and general base in the catalysis^26^. In *Aspergillus awamori* glucoamylase these (Glu179 and Glu400) and surrounding residues form a pocket accommodating the leaving glucosyl group^27^. Although the overall sequence identity between CgGDE and *Aspergillus awamori* glucoamylase is not significant (12%), the equivalent region in CgGDE forms an almost identical pocket, and Asp1241 and Glu1492 in it occupy equivalent locations as *Aspergillus awamori* glucoamylase residues Glu179 and Glu400 (Fig. 3b).

This homology suggests the C-terminal region of CgGDE catalyses a GC reaction similar to that of glucoamylase and other GH15 family members, and we have named this region the GC domain. To verify this, we generated the D1241A and E1492A mutants. Neither mutant has significant debranching activity (Fig. 3c and Supplementary Table 1). Their GT activity was not affected (Fig. 2c), indicating that their GC activity was lost. In ScGDE, mutations D1086N and D1147N were found to abolish the GC activity^23^. The Asp1086 equivalent in CgGDE (Asp1078) probably coordinates the leaving glucosyl group in the GC catalysis like the equivalent residue in glucoamylase (Asp55, Fig. 3b). As expected, the D1078N mutation in CgGDE also abolished the GC activity (Figs 2c and 3c and Supplementary Table 1). On the other hand, the Asp1147 equivalent in CgGDE (Asp1139) is located on the opposite side of the GC domain, and the equivalent mutation in CgGDE (D1139N) had little effect on the debranching activity (Fig. 3c, Table 1 and Supplementary Fig. 4a). Further studies are required to resolve this discrepancy.

### Intermediate translocation between the active sites

An important step in GDE-catalysed glycogen debranching is the translocation of the GT reaction product to the GC active site. It was proposed that this product does not dissociate from GDE, and that its translocation is facilitated by a spatial proximity between the GT and GC active sites on the same polypeptide^19^. However in CgGDE the GT and GC active sites are located >50 Å apart (Fig. 1b). To probe the mechanism of this intermediate translocation, we mixed a GT-defective mutant with a GC-defective mutant, and tested the mixture for debranching activity. These mutants alone have minimum activity (Fig. 3c). In contrast, all the mixtures we tested were as potent as the wild-type protein in catalysing the debranching reaction (Fig. 3d, Table 1 and Supplementary Fig. 4b). This indicates that CgGDE does not specifically favour the intra-molecular translocation of the GT reaction product.

### Substrate recognition by GDE

**Figure 4:**
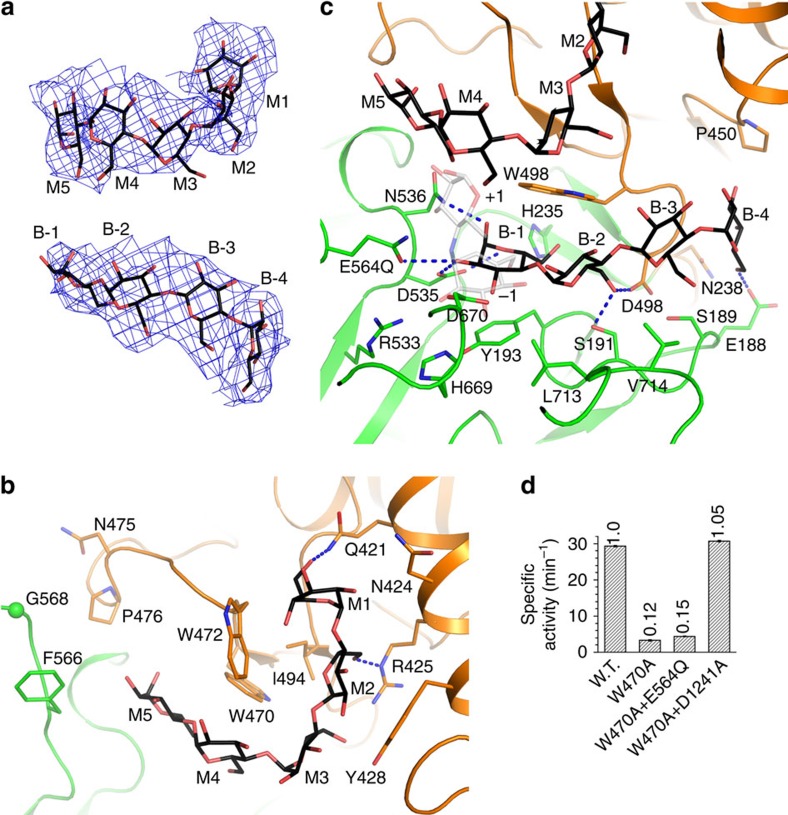
Substrate recognition by the GT domain active site. (**a**) Difference electron-density map for the oligosaccharides bound at the GT domain active site. The map shown here and in Fig. 5a was calculated before oligosaccharides were incorporated in the atomic model, and contoured at 2 σ. (**b**) Accommodation of oligosaccharide M by the GT domain active site. (**c**) Accommodation of oligosaccharide B by the GT domain active site. Part of the nearby oligosaccharide M is also shown. The +1 and −1 saccharide units of acarbose in the Taka-amylase A structure (PDB 7TAA, grey for the carbon atoms) is shown in partial transparency. They mimic the +1 and −1 residues in the substrate. (**d**) Specific debranching activities of the W470A mutant and its combinations with mutants possessing only the GT or the GC activities.

The maltopentaose complex structure revealed a number of oligosaccharides bound at the GT and GC active sites, providing insights into their substrate recognition. At the GT active site two oligosaccharides, M and B (for mainchain and branch, respectively; see below), can be modelled into the electron densities, which contains 5 and 4 residues, respectively (Fig. 4a and Supplementary Fig. 5a). Oligosaccharide M interacts with subdomain B. Its concave face interacts with Trp470, Trp472 and Ile494, and its non-reducing end forms additional interactions with Gln421, Asn424, Arg425 and Tyr428 (Fig. 4b). Oligosaccharide B is buried deep into a cleft between subdomains A and B. Its B-1 residue is accommodated by the equivalent of the −1 subsite in GH13 members, but the positions of this residue and the −1 residue in the Taka-amylase A substrate^28^ are somewhat different (Fig. 2b). This is probably due to the lack of additional residues at its reducing end in the current structure. Residues B-2 to B-4 form extensive interactions with Glu188, Ser189, Ser191, Asn238, Pro450, Trp496, Asp498, Leu713 and Val714 (Fig. 4c). Structural comparison with Taka-amylase A structure^28^ indicates that a glucose residue can fit into the space between residues B-1 and M5 (Fig. 4c), suggesting the GT substrate mainchain and branch bind at where oligosaccharides M and B bind, respectively. Such substrate accommodation presents the glycosidic bond to be cleaved (between the last and second to last residues in the branch) to the GT active centre (Supplementary Fig. 5b). A cleft formed by residues Asn475, Pro476, Phe566 and Gly568 at the reducing end of oligosaccharide M might mediate additional interactions with the substrate mainchain (Fig. 4b).

To verify this model, we generated the W470A mutant. Consistent with a role of Trp470 in stabilizing with the GT substrate mainchain, our debranching assays revealed that this mutant has severely impaired GT activity. Its drastically reduced debranching activity can be restored by supplementing the GT activity, but not by supplementing the GC activity (Fig. 4d, Table 1 and Supplementary Fig. 5c).

**Figure 5:**
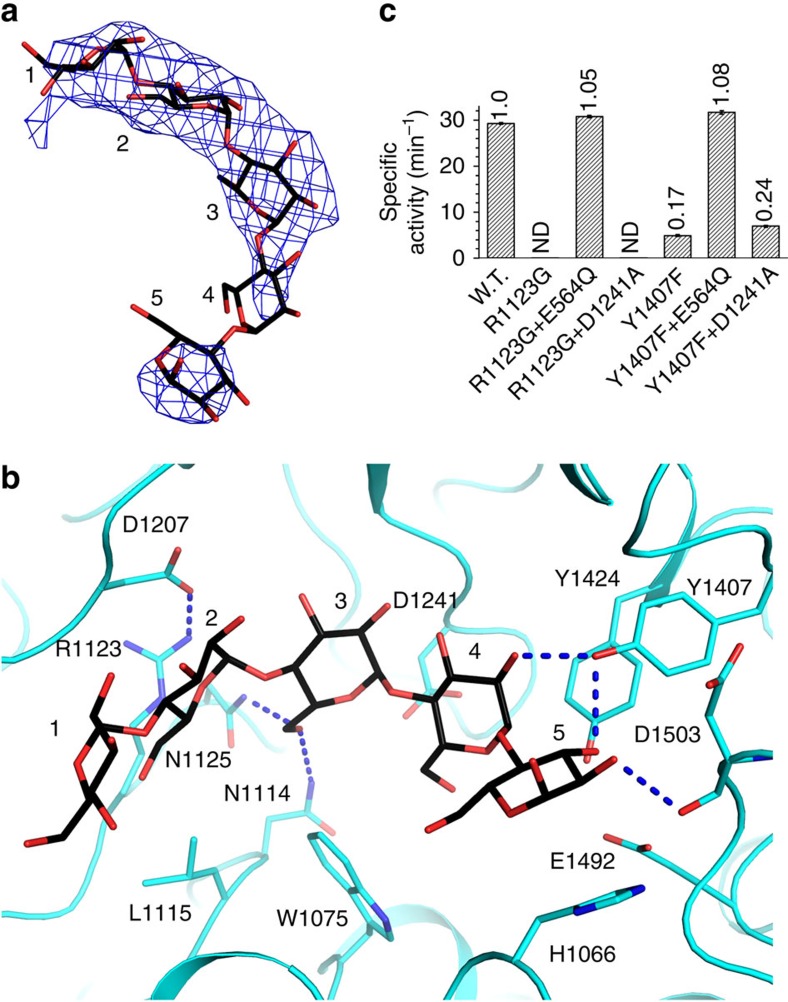
Substrate recognition by the GC domain active site. (**a**) Difference electron-density map for the oligosaccharide bound at the GC domain active site. (**b**) Accommodation of this oligosaccharide by the GC domain. (**c**) Specific debranching activities of CgGDE mutants R1123G and Y1407F, and their combinations with mutants possessing only the GT or GC activities.

At the GC domain active site an oligosaccharide with five residues can be fitted into the electron densities (Fig. 5a and Supplementary Fig. 5d). It binds into a cleft, with residues 2–3 interacting with Asn1114, Leu1115, Arg1123, Asn1125 and Asp1207, residues 4–5 interacting with His1066, Trp1075, Tyr1407, Tyr1424 and Asp1503, and the C6 hydroxyl of its residue 4 pointing towards the active-site pocket. This suggests that the cleft and the active-site pocket accommodate the GC substrate mainchain and its single-residue branch, respectively. This substrate accommodation presents the glycosidic bond to be cleaved (between the mainchain and the branch) to the GC active centre (Supplementary Fig. 5e). It indicates that the GC active site recognizes multiple residues in its substrate mainchain flanking the branch point, consistent with a study mapping the porcine GDE GC active-site structure^29^.

To verify this model, we generated the R1123G and Y1407F mutants. The Arg1123 side chain is expected to interact with the GC substrate mainchain, and forms a salt bridge with Asp1207 that probably stabilizes the structure of the mainchain-binding cleft. Consistently, the R1123G mutant completely lost the GC activity. No debranching activity can be observed for it or its combination with a mutant possessing only the GT activity, whereas supplementing it with a mutant possessing only the GC activity fully restored this activity (Fig. 5c, Table 1 and Supplementary Fig. 5f). The equivalent mutation in human GDE (R1147G) has the same effect^30^,^31^. The Y1407F mutation removes a hydroxyl group that is expected to form hydrogen bonds with the substrate mainchain (Fig. 5b). It also caused a severe reduction in GC activity (Fig. 5c, Table 1 and Supplementary Fig. 5g).

The structures of the GT and GC active sites indicate that they are highly selective towards substrate branch length. Modelling a sixth residue in the GT substrate branch creates steric clashes with the protein (Supplementary Fig. 6), indicating that the GT domain selects substrates with branches containing five or fewer residues. This is consistent with a study that probed the porcine GDE GT active-site structure^32^. The branch-binding pocket at the GC active site is quite shallow and cannot accommodate more than one residue (Supplementary Fig. 5e), enabling the GC domain to select single-residue branches.

### Additional contacts with glycogen

**Figure 6:**
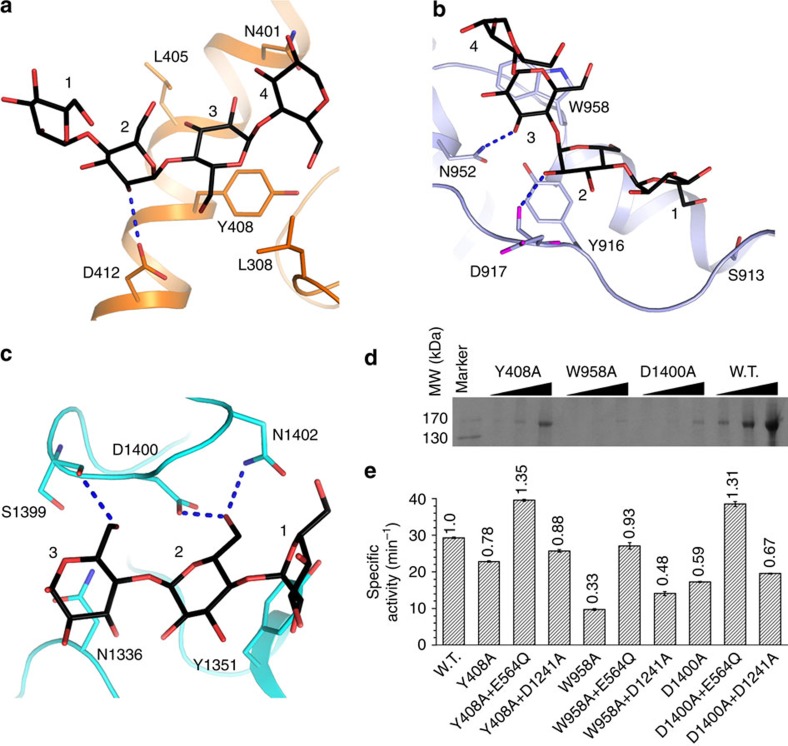
Additional contacts of CgGDE with glycogen. (**a**–**c**) Structures of the additional oligosaccharide-binding sites in GT subdomain B (**a**), domains M2 (**b**) and GC (**c**). (**d**) Mutations at the additional oligosaccharide-binding sites decrease the affinity to glycogen. CgGDE pulled-down by glycogen immobilized on concanavalin A agarose was analysed by SDS PAGE. (**e**) Specific debranching activities of CgGDE mutants Y408A, W958A, D1400A and their combinations with mutants possessing only the GT or GC activities.

The maltopentaose complex structure revealed additional bound oligosaccharides. An oligosaccharide with four residues can be fitted into the electron densities at GT subdomain B (Supplementary Fig. 7a). Its residue 2 hydrogen bonds with the Asp412 side chain, residue 3 stacks against the Tyr408 side chain and forms additional interactions with Leu308 and Leu405, and residue 4 interacts with Asn401 (Fig. 6a). The electron densities at domain M2 are consistent with an oligosaccharide with four residues (Supplementary Fig. 7b). Residue 1 interacts with Ser913, residues 2 and 3 interact with the Tyr916 and Trp958 side chains, respectively. Hydrogen bonds are formed between residue 2 and the Asp917 mainchain carbonyl, and between residue 3 and the Asn952 side chain (Fig. 6b). An oligosaccharide with three residues can be fitted into the electron densities at domain GC ∼20 Å away from the substrate-binding site (Supplementary Fig. 7c). Residue 1 interacts with Tyr1351, residue 2 hydrogen bonds with the Asp1400 and Asn1402 side chains, residue 3 hydrogen bonds with the Ser1399 mainchain carbonyl and interacts with Asn1336 (Fig. 6c).

GDE associates with glycogen^33^, and we investigated whether these oligosaccharide-binding sites contribute to this association. We introduced the Y408A, W958A and D1400A mutations that are expected to disrupt the observed protein–oligosaccharide interactions, and tested the mutants for glycogen binding. All these mutations severely reduced the association of CgGDE to glycogen (Fig. 6d), consistent with a role of these regions in glycogen association. To test whether this association has an effect on the GDE catalysis, we measured the activity of these mutants. All of them have significantly reduced debranching activity (Fig. 6e), which cannot be restored by supplementing them with the GT activity (Fig. 6e, Table 1 and Supplementary Fig. 7d–f). This suggests that their GC activities are impaired, and indicates the importance of glycogen engagement by these regions for the GC catalysis. Indeed, supplementing them with the GC activity restored their debranching activity. The higher debranching activity observed for the Y408A and D1400A mutants are probably due to their high residue GC activities (Fig. 6e, Table 1 and Supplementary Fig. 7d–f).

### Molecular insights into the disease-causing mutations

**Figure 7:**
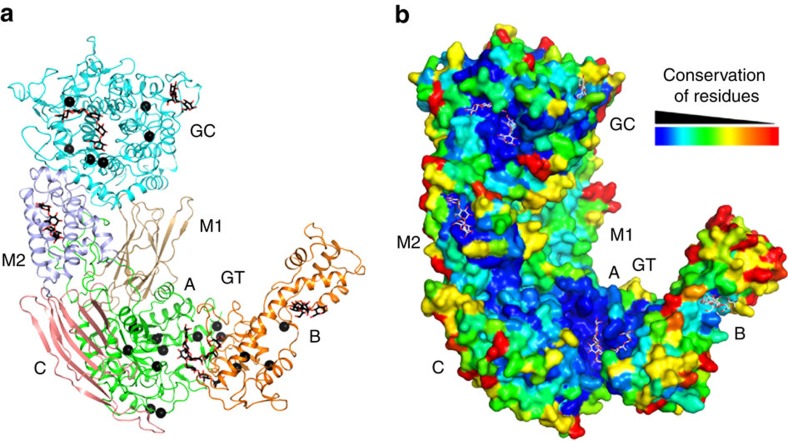
Disease-causing mutations and sequence conservation of GDE. (**a**) Disease-causing mutations of GDE. Missense mutations found in GSDIII patients are mapped onto the CgGDE structure, and represented by black spheres. (**b**) GDE residue conservation. The CgGDE is shown in surface representation, and coloured according to the conservation of individual residues. Bound oligosaccharides are shown in stick representation with their carbon atoms in white.

The CgGDE structure provides a basis to understand the disease-causing mutations associated with GSDIII. Mapping the reported missense mutations^34^,^35^,^36^ on the CgGDE structure revealed that they are located either on domain GT or GC (Fig. 7a and Supplementary Table 2). A number of the mutations are located at their substrate-binding sites, and are expected to disrupt substrate recruitment. These mutations include R428K, R524H and H626R (the equivalent positions in CgGDE are Pro450, Arg533 and His669, Fig. 4c) in the GT domain, and R1147G in the GC domain mentioned above. Several mutations located near the substrate-binding sites, including D215N and N219D in the GT domain, and G1087R and C1515R in the GC domain, likely have similar effects. Additional mutations might disrupt the folding and/or stability of GDE, indirectly affecting its activity. Mutations of this kind include L400P, L620P, G655R and G1448R. Decreased protein stability has been associated with the G1448R mutation^37^.

The majority of these mutations (16 out of 21) occur at positions invariant between human GDE and CgGDE. To verify our structural observations, we introduced equivalent mutations in CgGDE and measured the activities of the mutants (Supplementary Table 2). As expected, mutations at or near the GT substrate-binding site (D215N, N219D, R524H and H626R) caused severe reductions in GT activity, and mutations at or near the GC substrate-binding site (G1087R and R1147G) significantly impaired the GC activity. Consistent with the expected disruption of the folding/stability of the GT or GC domains, mutations L620P and G655R strongly inhibited the GT activity, and G1448R strongly inhibited the GC activity. In addition, mutations L400P and L620P caused substantial reductions in protein expression, consistent with decreased protein stability. Unexpectedly, we found that mutations G138E, R343W, R494H and A1120P did not have an effect on the CgGDE activity, and mutations R675W and D1364H only partially inhibited its activity. Among them, R343W (ref. 35), R494H (ref. 36), A1120P (ref. 38), R675W (ref. 39) and D1364H (ref. 40) were found in patients carrying additional mutations in their GDE gene, which might be responsible for the disease progression. The clinical relevance of the G138E mutation requires further study.

## Discussion

Our CgGDE structures gave significant insights into glycogen debranching in animals and fungi. The GT catalysis has long been thought to be similar to the reactions catalysed by GH13 family members. This is confirmed by the structure, which also revealed the GC catalysis is similar to that of GH15 family members. Our maltopentaose complex structure revealed highly specific substrate selectivity for the GT and GC active sites, which enables the GT active site to select glycogen phosphorylase-digested glycogen that contains four-residue branches^41^, and the GC active site to select the GT reaction product that has a single-residue branch. These substrate specificities therefore ensure concomitant changes of the GT and GC activities in response to changes in the glycogen phosphorylase activity, which is tightly regulated for highly controlled glycogen mobilization^2^. In addition to providing the extensive inter-domain interactions that stabilize the structure of the catalytic domains, the unique CgGDE tertiary structure also provides additional contacts for glycogen that mediate its recruitment to glycogen and regulate its activity. Its important regions, including the GT and GC active sites, and the additional glycogen-binding site on domain M2, are highly conserved among GDE from different organisms, indicating they have identical functions in these enzymes (Fig. 7b and Supplementary Fig. 2). The additional glycogen-binding sites on GT subdomain B and domain GC are not as conserved, and whether they have similar functions in GDE from other organisms requires further study.

Our data suggest that the GT reaction product completely dissociates from GDE before it is recruited to the GC active site. The additional glycogen-binding sites on GDE probably facilitate this recruitment, explaining their role in the GC catalysis. Similar cases have been reported for glycogen phosphorylase^42^, glycogen synthase^43^ and the glycogen dephosphorylase laforin^44^. In our *in vitro* assays, mutations at these regions do not appear to have significant impact on GT catalysis. However, a role of these regions in the GT catalysis cannot be ruled out. Their effects might become significant at conditions beyond the detection limits of our assays. Oligosaccharide binding to GDE has been reported to enhance its GT catalysis^45^,^46^, it remains to be seen if any of these regions play a role in this process. In addition, ubiquitination and stability of GDE are crucially regulated by its association with glycogen^37^, and it is likely that these regions are also involved in regulating the GDE turn over.

Introducing the missense mutations associated with GSDIII into CgGDE proved powerful in elucidating the molecular mechanism of their disease-causing effects. An interesting finding of these studies is that several of these mutations might be benign. However, one should be cautious in interpreting these data. The G138E mutation, for instance, is next to a one-residue deletion in the human GDE (Supplementary Fig. 2 and Supplementary Table 2), and might have different effects in CgGDE and human GDE.

In addition to glycogen metabolism, GDE also has important functions in degrading the polyglucosan bodies that accumulate in the cells of patients with the neurodegenerative Lafora disease^47^, regulating the energy master regulator AMP-activated protein kinase^48^ and suppressing cancer^49^,^50^. Our studies also provide a starting point to elucidate the mechanisms underlying these functions of GDE.

## Methods

### Protein expression and purification

The CgGDE gene was amplified from the *Candida glabrata* genome and inserted into the vector pET26b (Novagen). *Escherichia coli* BL21 Rosetta (DE3) cells transformed with this plasmid were cultured in LB medium supplemented with 34 mg l^−1^ kanamycin and 25 mg l^−1^ chloramphenicol, and induced with 0.3 mM Isopropyl β-D-1-thiogalactopyranoside (Bio Basic, Inc.) at 16 °C for 12 h. Cells were collected by centrifugation at 6,000*g* for 10 min, re-suspended in a buffer containing 20 mM Tris-HCl pH 7.5, 300 mM NaCl and 2 mM β-mercaptoethanol, and passed through a AH-2010 homogenizer (ATS Engineering, Inc.) three times. The lysate was cleared by centrifugation at 15,000*g* for 30 min, and soluble CgGDE was purified with nickel-nitrilotriacetic acid (Qiagen), ion-exchange (Hitrap Q HP, GE Healthcare) and size-exclusion (Sephacryl S300 HR, GE Healthcare) columns. Purified CgGDE was concentrated to 20 mg ml^−1^ in a buffer containing 20 mM Tris-HCl pH 7.5, 200 mM NaCl, 2 mM DTT and 5% glycerol, flash-cooled in liquid nitrogen and stored at −80 °C.

The SeMet-substituted CgGDE was produced by inhibiting the endogenous methionine synthesis of the expression host and supplementing it with SeMet^51^. The cells were cultured in M9 medium. One hour before induction, L-lysine (100 mg l^−1^), L-phenylalanine (100 mg l^−1^), L-threonine (100 mg l^−1^), L-isoleucine (50 mg l^−1^), L-leucine (50 mg l^−1^) and L-valine (50 mg ml^−1^) were added to the medium to inhibit the endogenous methionine synthesis, and DL-SeMet (50 mg ml^−1^) was supplemented. The protein was purified following the same protocol for the native protein, except that the DTT concentration was increased to 10 mM.

CgGDE mutations were generated with the QuikChange kit (Agilent Technologies) and verified by DNA sequencing. The expression and purification of the mutants followed the same protocol for the wild-type protein.

### Crystallization and structure determination

CgGDE crystals were obtained with the sitting-drop method at 20 °C. The reservoir liquid contained 10% poly(ethylene glycol) 5,000 monomethyl ether, 0.1 M Hepes pH 7.0 and 5% tacsimate pH 7.0. Before crystallization, 0.3 mM Tri(2-carboxyethyl)phosphine hydrochloride was added to the protein solution. To produce crystals of the CgGDE-maltopentaose complex, the protein solution was incubated with 100 mM maltopentaose (Sigma-Aldrich) for 1 h on ice before crystallization experiments. For data collection, crystals were equilibrated in the reservoir solution supplemented with 25% ethylene glycol for 30 s, flash-cooled and stored in liquid nitrogen.

Diffraction data were collected on an ADSC Q315 charge-coupled device detector at the Shanghai Synchrotron Radiation Facility beamline BL17U, at 100 K (Table 1). A MAD data set was collected on a ligand-free SeMet-substituted crystal, at three wavelengths near the Se K-edge (0.9793 Å, 0.9794 Å and 0.9537 Å). Another data set of the ligand-free crystal was collected on a native crystal at 1.0391 Å. The data set for the CgGDE-maltopentaose complex crystal was collected at 0.9792 Å. Diffraction data were scaled with mosflm^52^, integrated with scala^53^ and the intensities were converted to structure factors with ctruncate^54^.

The ligand-free CgGDE structure was determined by the MAD method using the anomalous signal of Se, with the phenix^55^ AutoSol pipeline. After the substructure search step, which found 61 sites (62 expected), the figure of merit was 0.47. Subsequent density modification gave an excellent electron-density map, which allowed building of the entire structure, with coot^56^ and O^57^. Using this structure as the search model, the CgGDE-maltopentaose complex structure was determined by the molecular replacement method with molrep^58^. Refinements were carried out with refmac^59^ and phenix^55^. An analysis with procheck^60^ indicates the ligand-free structure, refined against the native data set, has 84.3% residues in the most-favoured regions in the Ramachandran plot, 15.1% in the additionally allowed regions, 0.6% in the generously allowed regions and no residues in the disallowed regions. The refined CgGDE-maltopentaose complex structure has 84.8, 14.8, 0.3 and 0.1% residues in the most-favoured, additionally allowed, generously allowed and disallowed regions in the Ramachandran plot.

Structural homologues of CgGDE were searched with the Dali server^61^. Buried surface area between domains were calculated with areaimol^62^. Mosflm, scala, ctruncate, refmac, procheck and areaimol are programs in the ccp4 suite^63^.

### Dynamic light-scattering experiments

Dynamic light-scattering experiments were performed on a DynaPro Titan instrument (Wyatt Technologies) at 20 °C. CgGDE was measured at a concentration of 20 mg ml^−1^, in a buffer containing 20 mM Tris pH 7.5, 200 mM NaCl and 2 mM DTT. Data were analysed with the DYNAMICS V6 software (Wyatt Technologies).

### GDE activity assays

The GT activity was assayed by following the production of additional oligosaccharides from maltopentaose^16^. The reaction mixture contained 50 mM citric acid pH 6.5, 20 mM maltopentaose and 10 μM CgGDE. The reaction was allowed to proceed at 37 °C for 12 h and was terminated by heating at 100 °C for 5 min. After removing the precipitated protein by centrifugation, the reaction mixture was dried with an SPD121P speed vacuum concentrator (Thermo Scientific), re-dissolved in methanol and spotted on a HSGF254 thin-layer-chromatography plate (Yantai Chemical Research Institute) which was activated by heating at 110 °C for 1 h. The plate was put into a thin-layer-chromatography chamber containing n-butanol, ethanol and water in a 5:5:3 ratio, and the chromatography was developed at room temperature for 2 h. For visualization, the dried plate was dipped into an aniline-diphenylamine solution (1.8% aniline, 1.8% diphenylamine, 7.6% phosphoric acid and 89% acetone), air dried and baked at 110 °C for 10 min.

The overall GDE activity was assayed by following the glucose produced from glycogen phosphorylase-digested glycogen. Glycogen phosphorylase-digested glycogen was produced as described^12^. Briefly, 1 g of oyster glycogen (Sigma) and 200 U of rabbit muscle glycogen phosphorylase-a (Sigma) were dissolved in 20 ml digestion buffer (0.2 M phosphate, pH 6.8), and were dialyzed against a large volume of digestion buffer at 37 °C overnight. Afterwards glycogen phosphorylase was precipitated by heating at 95 °C for 30 min and removed by centrifugation. Glycogen phosphorylase-digested glycogen was subsequently precipitated by adding 40 ml of ethanol and collected by centrifugation. Glucose production was coupled to NAD^+^ reduction with hexokinase and glucose-6-phosphate dehydrogenase^64^, and the resulting absorption change at 340 nm was monitored on an ultraspec 2100 pro spectrophotometer (GE Healthcare). The reaction mixture contained 16.7 mM citric acid pH 6.5, various amounts of glycogen phosphorylase-digested glycogen, 1.67 × of the hexokinase/glucose-6-phosphate dehydrogenase reaction mix (1.67 U ml^−1^ hexokinase, 1.67 U ml^−1^ glucose-6-phosphate dehydrogenase, 2.5 mM NAD^+^ and 1.67 mM ATP; Sigma-Aldrich) and 0.33 μM CgGDE. For reactions containing two CgGDE mutants, 0.33 μM of each mutant was added.

The interaction between glycogen and CgGDE was assayed by pull-down experiments using glycogen immobilized on concanavalin A (conA) agarose beads^65^. 100 μl of conA agarose beads (Sigma-Aldrich) pre-equilibrated in the binding buffer (67 mM Hepes pH 7.5, 0.2 mM CaCl_2_, 10 mM MgCl_2_, 1 mM MnCl_2_) were incubated with 30 mg of glycogen (Sigma-Aldrich) at 4 °C for 1 h. After washing three times with the binding buffer, the charged beads were incubated with increasing amounts of CgGDE (25, 100 and 400 μg) at 4 °C for 1 h. Unbound CgGDE was removed by washing three times with the binding buffer, and the amount of bound CgGDE was revealed by SDS PAGE analysis with Coomassie blue staining. The assay was repeated three times with similar results. A representative uncropped scan of the SDS PAGE is presented in Supplementary Fig. 8.

## Additional information

**Accession codes:** The structure factors and coordinates for the structures of CgGDE and its complex with maltopentaose have been deposited into the Protein Data Bank, with accession codes 5D06 and 5D0F, respectively.

**How to cite this article:** Zhai, L. *et al.* Crystal structure of glycogen debranching enzyme and insights into its catalysis and disease-causing mutations. *Nat. Commun.* 7:11229 doi: 10.1038/ncomms11229 (2016).

## Supplementary Material

Supplementary Information Supplementary Figures 1-8 and Supplementary Tables 1-2.

## Figures and Tables

**Table 1:**
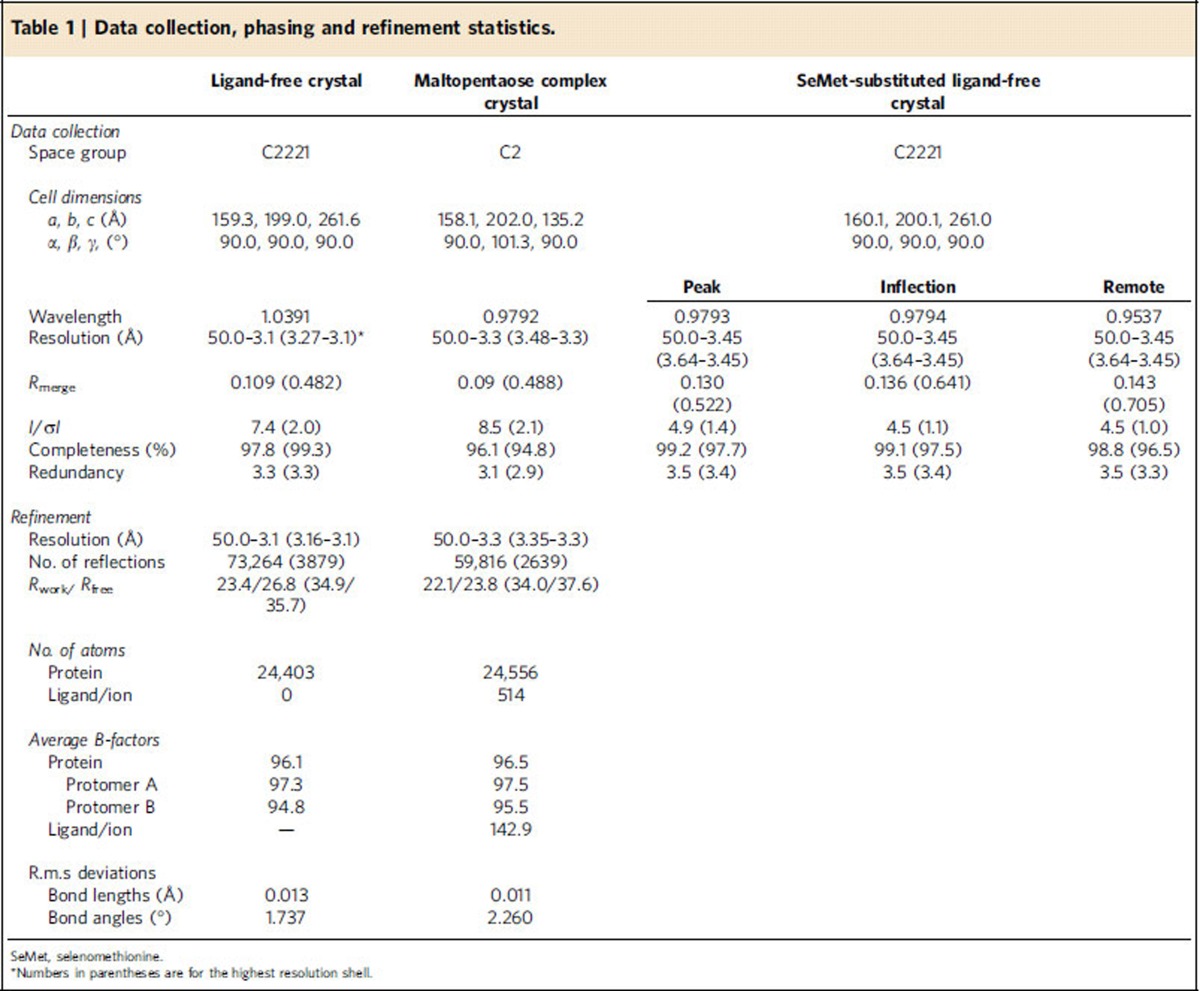
Data collection, phasing and refinement statistics.
