# Supplementary material for: Crystal structure of glycogen debranching enzyme and insights into its catalysis and disease-causing mutations
Source: Nat Commun. 2016 Apr 18;7:11229. doi: 10.1038/ncomms11229 (PMC4837477; doi:10.1038/ncomms11229)
Supplement: Supplementary Information — Supplementary Figures 1-8 and Supplementary Tables 1-2. [file ncomms11229-s1.pdf]

SUPPLEMENTARY FIGURES

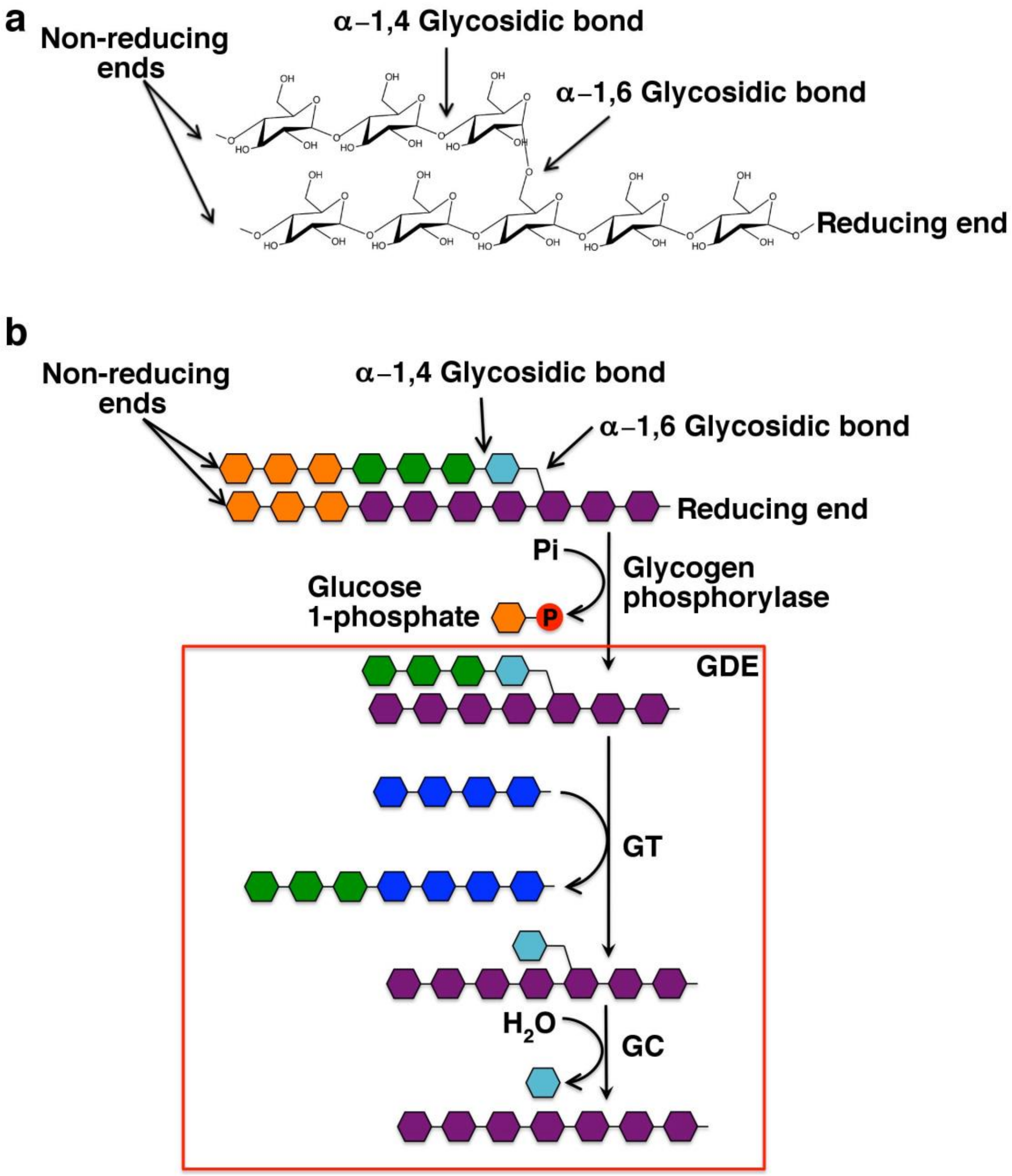

Supplementary Figure 1 Glycogen degradation by glycogen phosphorylase and GDE. (a) Chemical structure of glycogen. A few residues near a branch point are shown. (b) Glycogen degradation by glycogen phosphorylase and GDE. Glucose residues are represented by hexagons. The orange ones are removed by glycogen phosphorylase, green ones by the GT activity of GDE, and the one in cyan is removed by the GC activity. The debranched chain (magenta) can then be further degraded by

glycogen phosphorylase.

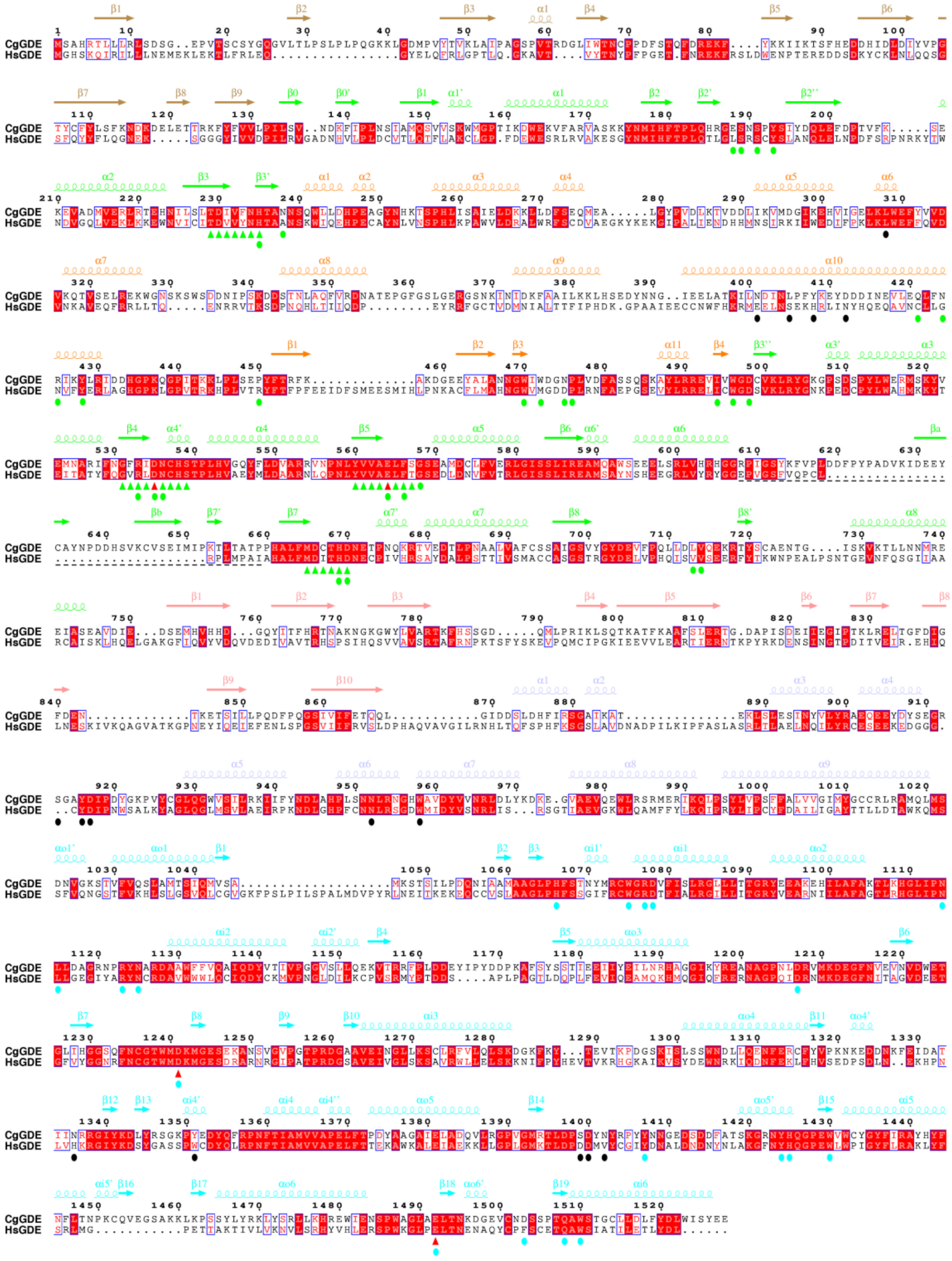

Supplementary Figure 2 Sequence alignment of CgGDE and human GDE (HsGDE). Residue numbers and secondary structural elements are indicated for CgGDE, and the latter are colored as in Fig. 1a. Secondary structure elements α1-8 and β1-8 in GT subdomain A form a TIM barrel, and αo1-6 and αi1-6 in domain GC form a $(\alpha/\alpha)_6$

barrel. Catalytic residues are labeled with red triangles, additional residues in CSRI-IV in domain GT are labeled with green triangles. Dots of different colors indicate residues that interact with substrates in the GT domain (green), or the GC domain (cyan), or provide additional contacts with glycogen (black). The dashed line indicates the α6-β7 loop in GT subdomain A, and the strands of the β hairpin formed in this region in protomer B in the ligand-free crystal are indicated by βa and βb.

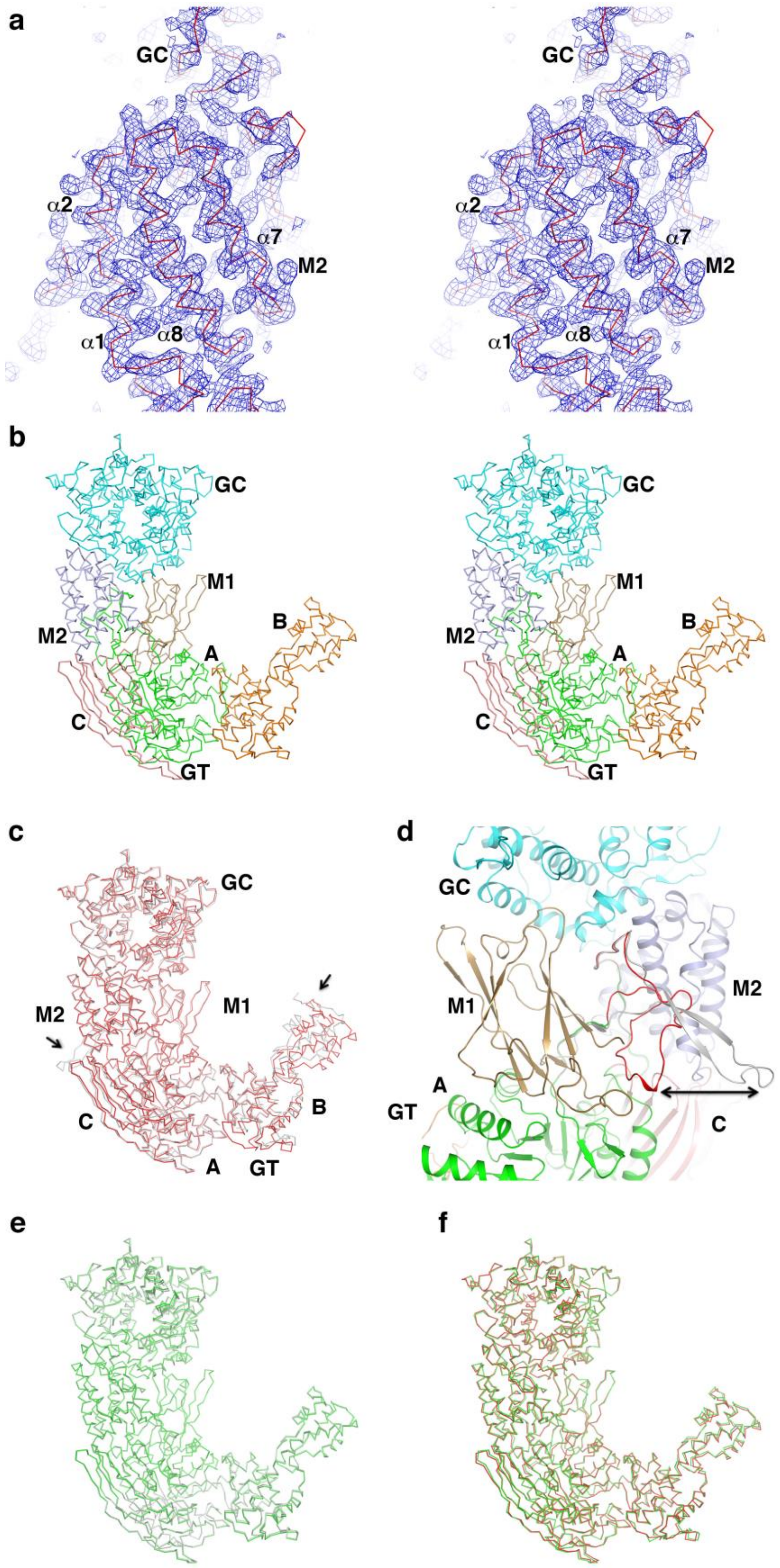

Supplementary Figure 3 Structure of CgGDE. (a) Stereo view of representative experimental electron density generated by the multiple anomalous diffraction

experiment. Electron density generated after the density modification step in the phenix AutoSol pipeline is shown in blue and contoured at 1 σ. The Cα trace of the built model is shown in red. Domain names and secondary structure elements are indicated. (b) Stereo view of the CgGDE structure. The structure of protomer A in the ligand-free crystal is shown. (c) Superimposition of protomers A (red) and B (gray) in the ligand-free crystal. The arrows indicate regions with the largest conformational differences. (d) The large conformational difference of residues 629-651 in the ligand-free crystal. The double-headed arrow indicates the conformational difference between protomers A and B (gray). Residues 619-651 that are absent in the human GDE are highlighted in red in protomer A. (e) Superimposition of protomers A (green) and B (gray) in the maltopentaose complex crystal. (f) Superimposition of protomers A in the ligand-free (red) and maltopentaose complex (green) crystals.

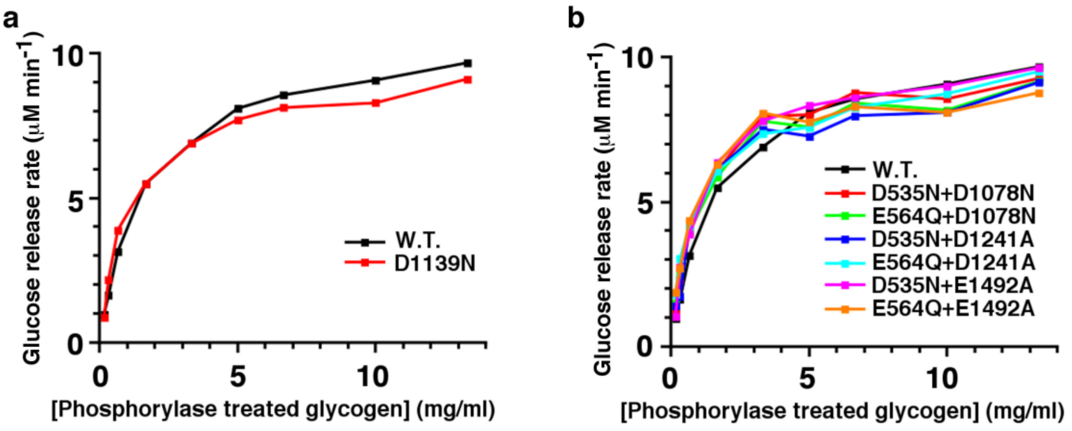

Supplementary Figure 4 Debranching activities of CgGDE mutants and their combinations. (a) Debranching reaction rates of the D1139N mutant at different substrate concentrations. (b) Debranching reaction rates of combinations of GT-defective and GC-defective mutants at different substrate concentrations. In both panels reaction rates of the wild type CgGDE is shown for reference.

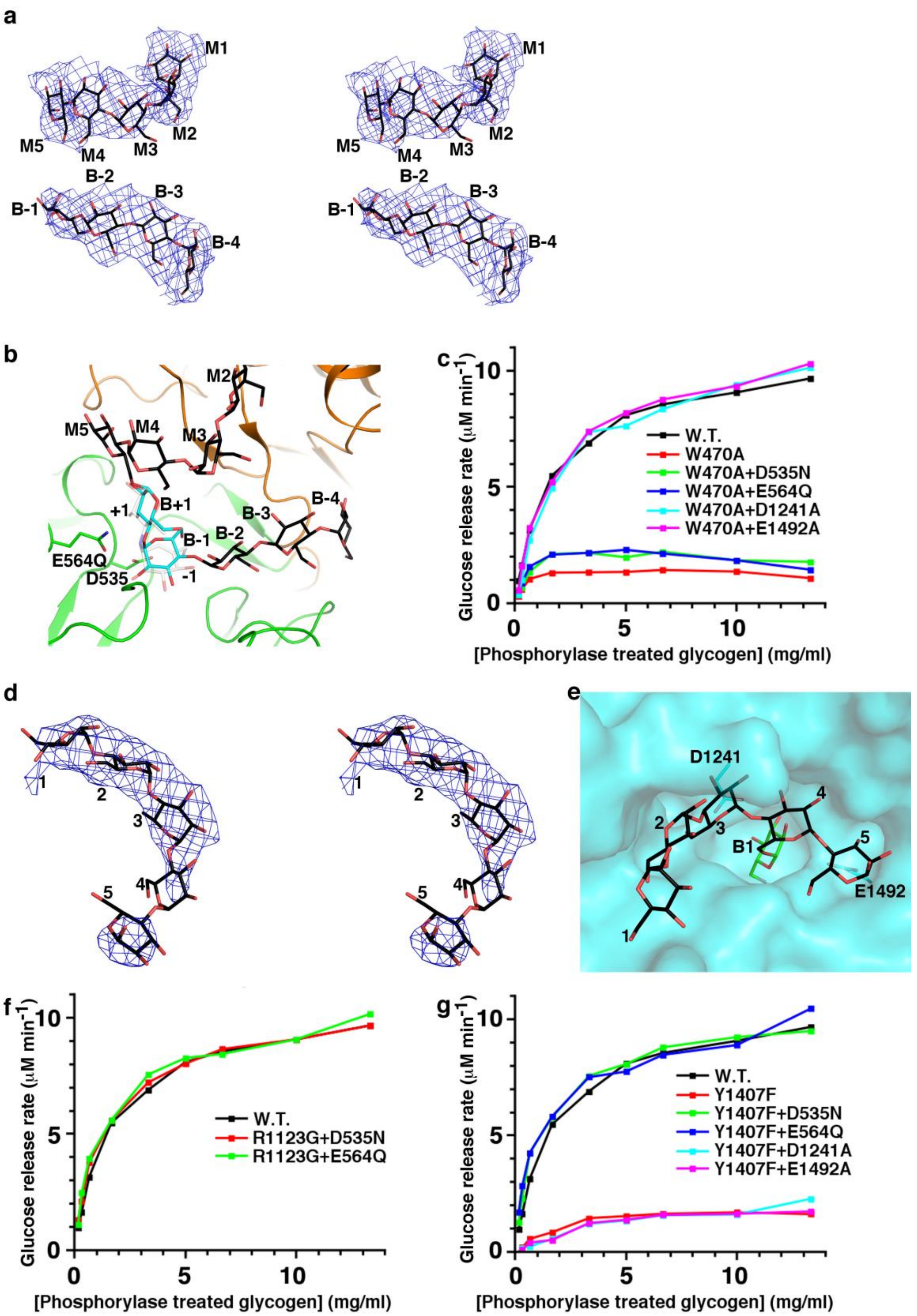

Supplementary Figure 5 Substrate recognition by the GT and GC domains. (a) Stereo view of the difference density map for oligosaccharides bound at the GT active site. The map shown here and in panel d, as well as in Supplementary Figs. 7a-c was

calculated before oligosaccharides were incorporated in the model, and contoured at 2 σ. (b) Structural model of substrate accommodation by the GT domain. Residues B+1 and B-1 in the branch (cyan for the carbon atoms) are modeled based on the positions of the +1 and -1 saccharide units in acarbose in the Taka-amylase A structure (PDB 7TAA, gray for the carbon atoms). Positions of other residues are identical to equivalent ones in the maltopentaose complex structure. Catalytic residues are highlighted. (c) Debranching reaction rates of the W470A mutant and its combinations with mutants possessing only the GT or GC activity. (d) Stereo view of the difference density map for the oligosaccharide bound at the GC active site. (e) Structural model of substrate accommodation by the GC domain. The protein surface and the catalytic residue side chains are shown. Positions of the substrate mainchain residues are identical to the equivalent ones in the maltopentaose complex structure. A single-residue branch (B1, green for the carbon atoms) is modeled into the active site pocket. (f) and (g) Debranching reaction rates of the R1123G (f) and Y1407F (g) mutants and their combinations with mutants possessing only the GT or GC activity.

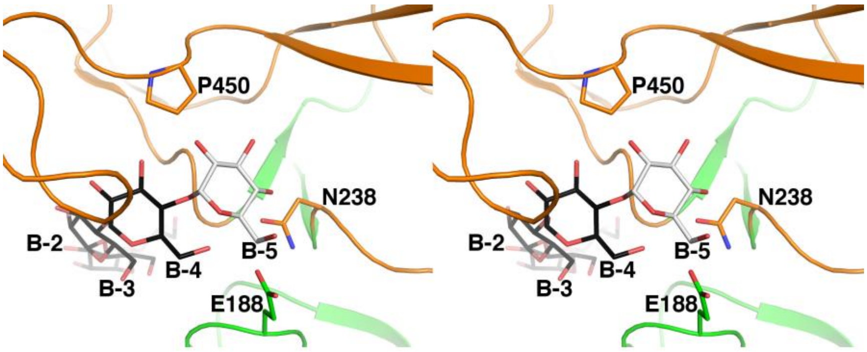

Supplementary Figure 6 Structural model of a 6-residue glycogen branch bound at the GT domain active site. The structure is shown in stereo. The modeled sixth residue in the branch (B-5, gray for the carbon atoms) forms steric clashes with the protein, indicating that long branches do not bind favorably.

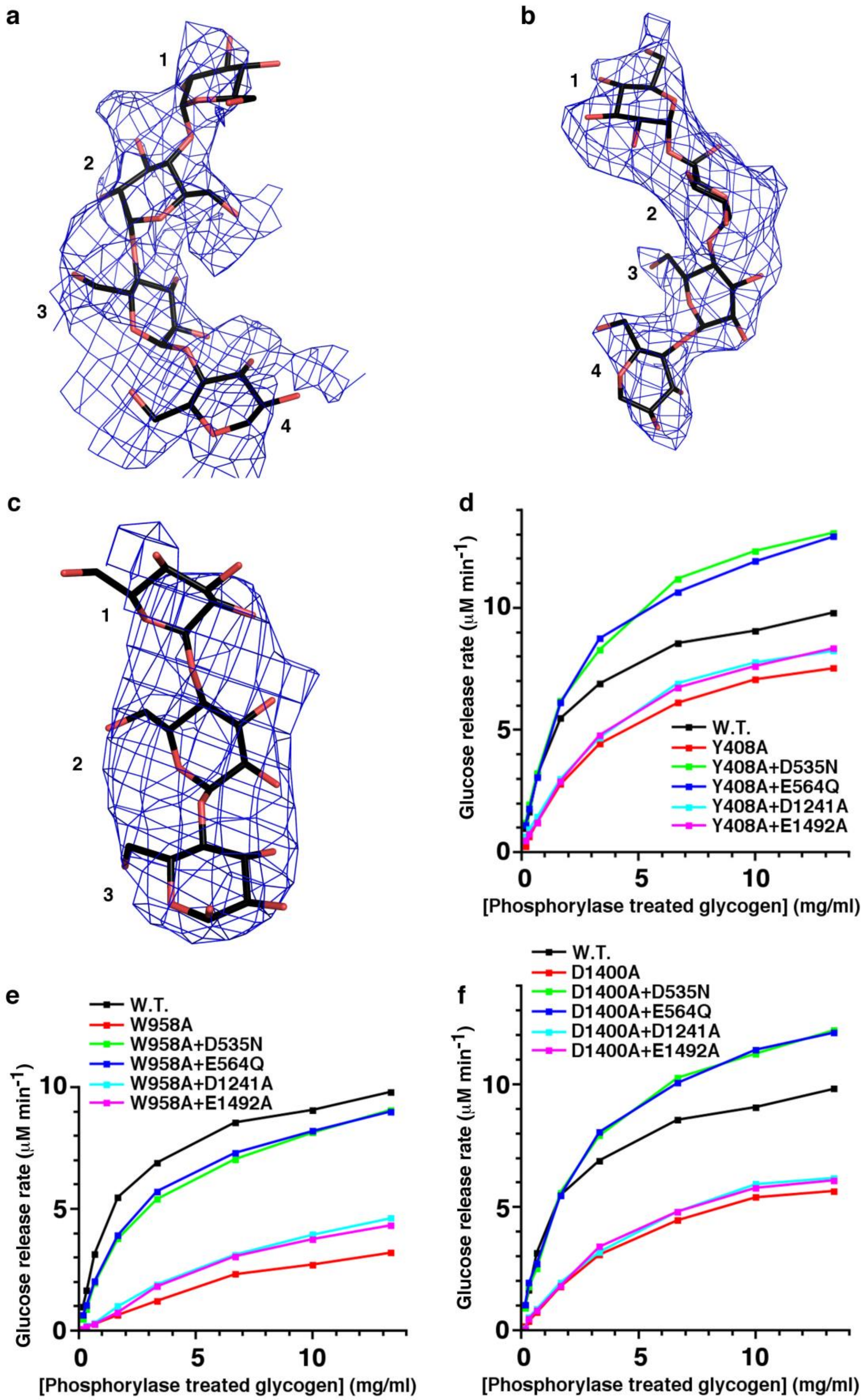

Supplementary Figure 7 Additional binding sites for glycogen. (a)-(c) Difference

electron densities for oligosaccharides bound at additional sites in the GT subdomain B (a), domains M2 (b) and GC (c). (d)-(f) Debranching reaction rates for the CgGDE mutants Y408A (d), W958A (e), and D1400A (f), and their combinations with mutants possessing only the GT or GC activity.

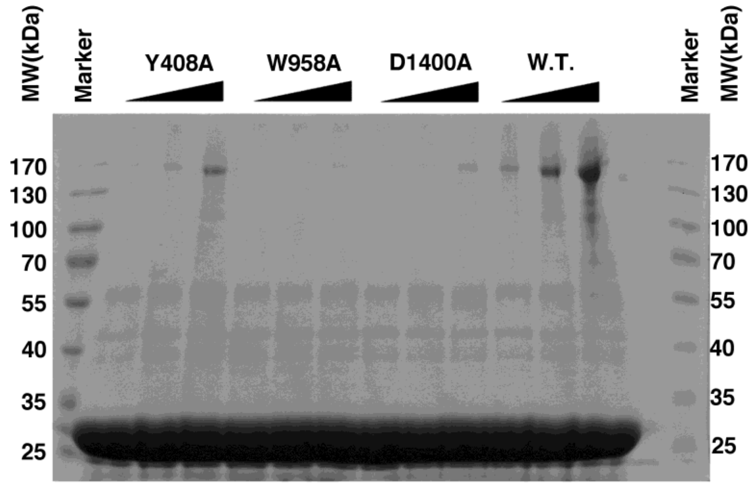

Supplementary Figure 8 SDS PAGE analysis of CgGDE or its mutants pulled down by glycogen immobilized on concanavalin A agarose. A representative uncropped scan is presented.

SUPPLEMENTARY TABLES

Supplementary Table 1 Specific activities of CgGDE and its mutants

	Specific activity (min^{-1})		Specific activity (min^{-1})
W.T.	29.30±0.20 (1.0)[1]	R1123G	ND
D535N	0.62±0.06 (0.021)	R1123G+E564Q	30.79±0.27 (1.05)
E564Q	0.39±0.22 (0.013)	R1123G+D1241A	ND
D1078N	0.58±0.10 (0.020)	Y1407F	4.92±0.22 (0.17)
D1139N	27.61±0.15 (0.94)	Y1407F+E564Q	31.68±0.40 (1.08)
D1241A	0.52±0.06 (0.018)	Y1407F+D1241A	6.91±0.21 (0.24)
E1492A	ND	Y408A	22.83±0.20 (0.78)
D535N+D1078N	28.06±0.19 (0.96)	Y408A+E564Q	39.53±0.27 (1.35)
D535N+D1241A	27.66±0.68 (0.94)	Y408A+D1241A	25.72±0.35 (0.88)
D535N+E1492A	29.18±0.18 (1.00)	W958A	9.73±0.25 (0.33)
E564Q+D1078N	27.77±0.59 (0.95)	W958A+E564Q	27.10±0.87 (0.93)
E564Q+D1241A	28.79±0.08 (0.98)	W958A+D1241A	14.06±0.55 (0.48)
E564Q+E1492A	26.57±0.17 (0.91)	D1400A	17.26±0.16 (0.59)
W470A	3.28±0.20 (0.12)	D1400A+E564Q	38.52±0.67 (1.31)
W470A+E564Q	4.38±0.10 (0.15)	D1400A+D1241A	19.52±0.12 (0.67)
W470A+D1241A	30.69±1.22 (1.05)		

[1]Numbers in parentheses are ratios to the wild type value.

Supplementary Table 2 Miss-sense mutations associated with GSDIII.

**Mutation**	**Equivalent position in CgGDE**	**Structural Observations**	**Specific activity (min^{-1})[1, 2]**
G138E	G157	On the surface of the GT subdomain A, precedes helix α1 and next to a one-residue deletion in human GDE. Close to the R675W mutation.	29.9±0.4 (1.02)
D215N	D230	Near the B-1 residue in the GT substrate, hydrogen bonds with Tyr193, probably affects substrate binding. Close to the R524H mutation.	1.3±0.2 (0.043)<br>1.79±0.03 (0.061)<br>25.4±0.2 (0.87)
N219D	N234	Near the B-1 residue in the GT substrate, probably	1.5±0.2 (0.052)

		affects substrate binding.	1.9±0.1 (0.065)<br>29.4±0.6 (1.00)
C234R	A249	In GT subdomain B at the interface with subdomain A, in a hydrophobic environment, probably affects protein folding/stability. Close to the R494H mutation.	
A253P	K268	Located at the end of helix α3 in domain GT subdomain B, probably affects protein folding/stability.	
R343W	R366	On the surface of GT subdomain B.	29.2±0.3 (1.00)
L400P	L422	In helix α10 in GT subdomain B, probably affects protein folding/stability.	[3]
R428K	P450	In the GT domain substrate binding site, interacts with residue B-4 in the substrate, probably affects substrate binding.	
R494H	R503	In GT subdomain A, at the interface with subdomain B. Close to the C234R mutation.	32.1±0.5 (1.10)
R524H	R533	In the GT domain substrate binding site, interacts with residue B-1 in the substrate, probably affects substrate binding.	1.8±0.1 (0.062)<br>2.1±0.3 (0.070)<br>27.5±1.2 (0.94)
L620P	L663	In the interior of GT subdomain A, on strand β7, probably affects protein folding/stability. Close to the G655R mutation.	7.6±0.2 (0.26)[4]<br>11.2±0.1 (0.38)<br>21.3±0.8 (0.72)
H626R	H669	In the GT domain substrate binding site, interacts with residue B-1 in the substrate, probably affects substrate binding.	1.8±0.1 (0.060)<br>2.4±0.2 (0.082)<br>25.40±0.07 (0.870)
G655R	G698	In the interior of GT subdomain A in a hydrophobic environment, probably affects protein	2.66±006 (0.090)<br>3.3±0.2 (0.113)

		folding/stability. Close to the L620P mutation.	28.65±0.08 (0.978)
R675W	R718	On the surface of GT subdomain A, forms a salt bridge with Glu716, probably affects protein folding/stability. Close to the G138E mutation.	19.2±0.6 (0.656)<br>29.1±0.6 (0.994)<br>29.4±0.2 (1.00)
R864P	-	Located between domains GT and M2, in an 11-residue stretch that exists in human GDE but not in CgGDE.	
G1087R	G1063	Near the GC domain active site pocket that accommodates the single-residue glycogen branch, probably affects substrate binding. Close to the C1515R mutation.	0.6±0.2 (0.020)<br>29.8±0.2 (1.02)<br>1.1±0.2 (0.038)
A1120P	A1096	In helix αO2 in domain GC.	31.7±0.6 (1.08)
R1147G	R1123	In the GC domain substrate binding site, forms a salt bridge with Asp1207, probably affects substrate binding.	ND<br>30.8±0.3 (1.05)<br>ND
D1364H	D1343	In the interior of domain GC, forms a salt bridge with Arg1259, probably affects protein folding/stability.	17.4±0.4 (0.59)<br>37.2±0.8 (1.27)<br>19.7±0.2 (0.67)
G1448R	G1427	In the interior of domain GC, in a hydrophobic environment, probably affects protein folding/stability.	1.5±0.2 (0.052)<br>25.6±0.3 (0.88)<br>2.5±0.1 (0.085)
C1515R	S1505	Near the GC domain substrate binding site, probably affects substrate binding. Close to the G1087R mutation.	

[1]Specific debranching activities were measured for the equivalent CgGDE mutants. Numbers in parentheses are ratios to the wild type value. A few mutations that occur at positions not conserved between the human GDE and CgGDE were not included in

this study. Average values and standard deviations from triplicate experiments are shown.

[2]For mutants with substantially decreased activities, the impact of the mutation on their GT and GC activities were assessed by measuring the activity of their mixture with mutants containing only GC (E564Q) or GT (D1241A) activities. These measurements were shown on the second and third lines, respectively.

[3]We were not able to purify the equivalent CgGDE L422P mutant due to extremely low protein expression.

[4]The protein yield of the equivalent CgGDE L663P mutant is significantly lower than that of other mutants and wild-type CgGDE in our purification.
